# Fingerprint-Based Machine Learning for SARS-CoV‑2 and MERS-CoV *M* ^*pro*^ Inhibition: Highlighting the Potential of Bayesian Neural Networks

**DOI:** 10.1021/acs.jcim.5c02014

**Published:** 2025-12-10

**Authors:** Niklas Piet Doering, Valerij Talagayev, Sijie Liu, Gerhard Wolber

**Affiliations:** Department of Biology, Chemistry and Pharmacy, Institute of Pharmacy, Molecular Design Group, Freie Universität Berlin, Königin-Luisestr. 2 + 4, 14195 Berlin, Germany

## Abstract

Severe acute respiratory syndrome coronavirus 2 (SARS-CoV-2) and Middle East respiratory syndrome coronavirus (MERS-CoV) are two important targets in current drug discovery, mainly due to the COVID-19 pandemic and the MERS-CoV outbreaks in recent years. An important target of both SARS-CoV-2 and MERS-CoV is the main protease (*M* ^*pro*^). Recently, the ASAP Discovery Consortium focused on the acceleration of *M* ^*pro*^ inhibitors with a part of this initiative being an open blind challenge in collaboration with Valence lab using the Polaris platform, where data sets of previously undisclosed inhibitors of SARS-CoV-2 *M* ^*pro*^ and MERS-CoV *M* ^*pro*^ were shared with researchers, to allow the development of machine learning and deep learning models for the prediction of the potency. We used this opportunity to evaluate and compare traditional machine learning models consisting of a random forest (RF) and gradient boosting model (XGBoost) with a bayesian neural network (BNN) model. For this purpose, we created single task models for the predictions of each of the targets. The results obtained showed that the BNN model outperformed both traditional machine learning models for both targets, indicating that BNNs are a promising deep learning framework in low-data regimes.

## Graphical abstract

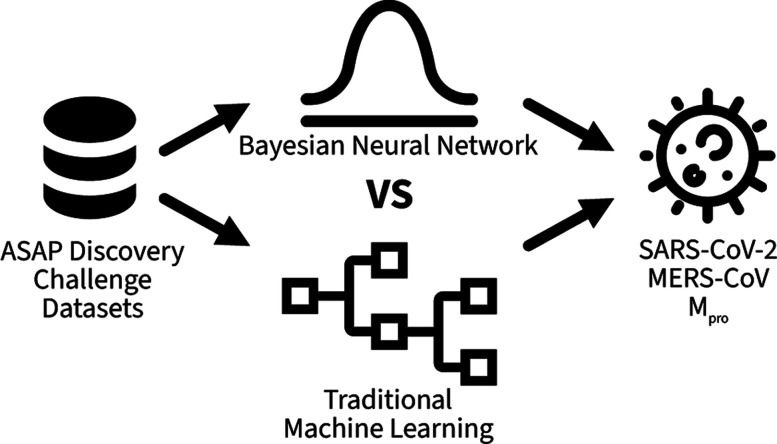

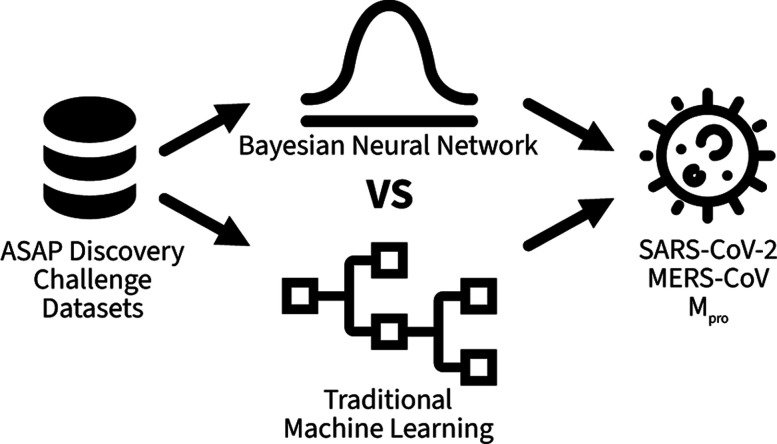

## Introduction

Severe acute respiratory syndrome coronavirus 2 (SARS-CoV-2) and Middle East respiratory syndrome coronavirus (MERS-CoV) are two highly pathogenic members of the *Coronaviridae* family that have caused significant global health concerns in recent times. With SARS-CoV-2 being the virus that led to the COVID-19 pandemic beginning in late 2019,^1,2^ MERS-CoV emerged slightly earlier in 2012 with sporadic outbreaks mainly in the Middle East.^3^ Due to the rapid spread of especially SARS-CoV-2, many drug discovery efforts have been started to target these rather novel viruses.^4^ A target of great interest within these viruses is the main protease (*M* ^*pro*^), which plays an important role as a viral enzyme that cuts polyproteins into functional pieces needed for coronavirus replication.^5,6^ Recent efforts, such as those led by the ASAP Discovery Consortium, have focused on accelerating the discovery of broad-spectrum *M* ^*pro*^ inhibitors using a combination of structural biology, medicinal chemistry, and artificial intelligence.^7,8^ As part of this initiative, a blind challenge was launched leveraging the Polaris platform to host data sets,^9–11^ using previously undisclosed antiviral potency data obtained from the ASAP pan-coronavirus *M* ^*pro*^ program, including a preclinical candidate set to be disclosed.^12^ With the data set being withheld from disclosure to ensure a successful patent application this provided an opportunity for an open challenge, which would act as a real-world benchmark for evaluating predictive modeling strategies.^13^ This setting offered a valuable opportunity to explore different ML and DL methods in drug discovery.

When choosing a modeling strategy for ligand-based prediction, it is important to balance interpretability and robustness. Tree-based algorithms such as Random Forest (RF) and gradient boosting are common choices for small-to-medium chemical data sets, as they perform well with minimal feature preprocessing, handle diverse molecular descriptors, and provide interpretable feature importance measures.^14–16^ Deep learning (DL), in contrast, can capture more complex nonlinear structure–activity relationships (SAR), but its advantages typically require larger data sets.^17,18^ In small or noisy data sets, such as those encountered in early stage drug discovery, DL architectures often overfit and struggle to learn transferable representations, making additional strategies necessary to ensure reliable performance.^18,19^ Moreover, in early stage medicinal chemistry, quantifying predictive uncertainty is as important as achieving high accuracy, as overconfident yet incorrect predictions can lead to wasted synthesis and screening efforts.

A promising approach to address limited data availability in deep learning is the use of Bayesian neural networks (BNNs). While BNNs retain the layered architecture of conventional neural networks, they differ fundamentally in how they represent learned parameters. At its core, a Bayesian Neural Network shifts our perspective from learning weights as single values to learning probability distributions over them (Figure 1).^20–22^ As in this setting, the parameters and output are both considered to be random variables, according to Bayes’ theorem, we can compute the posterior distribution as^22^

1 $$p\left(w|X,Y\right)=\frac{p\left(Y|X,w\right)p\left(w\right)}{p\left(Y|X\right)}$$

2 wherep(w)isourpriorandp(Y|X,w)isthemodellikelihood.

**Figure 1:**
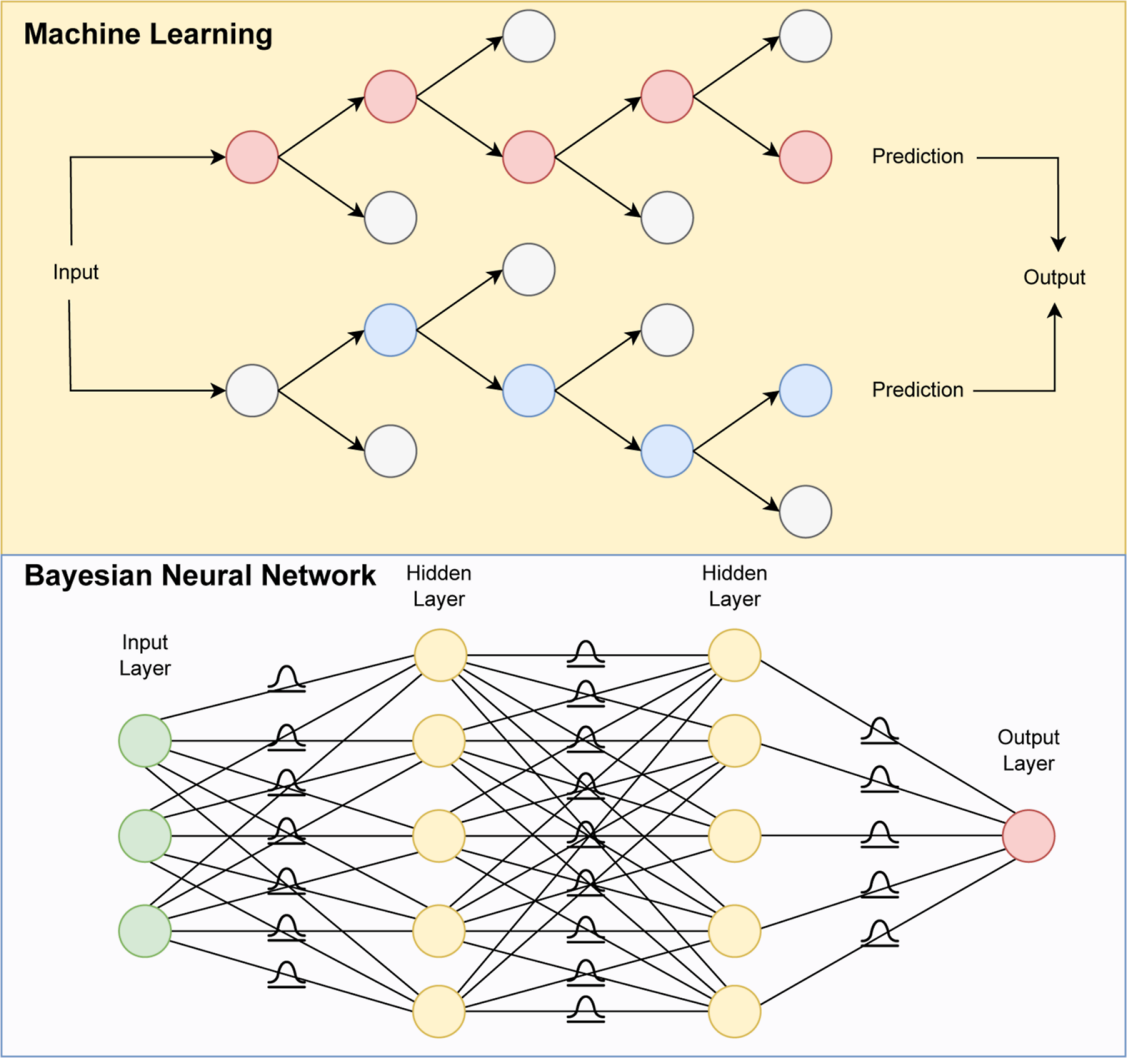
Schematic representation of machine learning and Bayesian neural network architectures. The machine learning (random forest) makes predictions by combining the outputs of many decision trees, its parameters are fixed once the model is trained. In contrast, the Bayesian neural network treats its parameters as probability distributions rather than fixed values.

For predictions on new inputs x*, we integrate over the entire posterior distribution of weights to obtain the predictive distribution^22^

3 $$p\left(y^{*}|x^{*},X,Y\right)=\int_{\Omega }p\left(y^{*}|x^{*},w\right)p\left(w|X,Y\right)dw$$

4 wherew∈Ω

However, this integral is intractable for neural networks because it ranges over a high-dimensional parameter space.^21^ Variational inference sidesteps this by approximating the true posterior *p*(*w*|*X*,*Y*) with a tractable distribution *q*_θ_(*w*) (typically a fully factorized Gaussian). Minimizing the Kullback–Leibler divergence between *q*_θ_(*w*) and *p*(*w*|*X*,*Y*) is equivalent to maximizing the evidence lower bound (ELBO) during training

5 $$L_{VI}\left(\theta \right)=\int_{\Omega }q_{\theta }\left(w\right)\log p\left(Y|X,w\right)dw-KL\left(q_{\theta }\left(w\right)∥p\left(w\right)\right)$$

The first term encourages good fit to the observed data, while the KL term acts as regularization, pulling the approximate posterior toward the prior.^22^ In essence, rather than committing to fixed weights, the model learns to express uncertainty through weight distributions.^21^

During inference, weight samples are drawn from the approximate posterior *q*_θ_(*w*), allowing the model to quantify predictive uncertainty. This probabilistic framework proves particularly valuable in data-limited scenarios typical of early stage drug discovery, where understanding prediction confidence is essential for informed decision-making.^23^

In this study, we aim to demonstrate the potential of BNNs in drug design scenarios characterized by limited data availability. Therefore, we compared a BNN-based deep learning approach^20,24^ with two widely used machine learning baselines: the gradient boosting framework XGBoost^25^ and Random Forest (RF).^26^ XGBoost builds an ensemble of decision trees sequentially, with each tree correcting the errors of its predecessors, whereas RF constructs trees in parallel and averages their outputs to reduce overfitting. Both methods perform well in ligand-based applications, particularly with structured inputs such as molecular fingerprints, including MACCS keys,^27,28^ path-based RDKit fingerprints,^27^ and circular ECFP/Morgan fingerprints.^27,29^

## Results

During the ASAP Discovery potency prediction challenge,^13^ we were required to predict the SARS-CoV-2 *M* ^*pro*^ and MERS-CoV *M* ^*pro*^ inhibition potency (pIC_50_ = −log_10_ of the molar IC_50_) of a set of molecules. We were given a training set, with known inhibition potency values, and a validation set, where the pIC_50_ values were not disclosed during the challenge. The training set consisted of 842 molecules for the SARS-CoV-2 *M* ^*pro*^ data points and 901 molecules for the MERS-CoV *M* ^*pro*^ data points, while the validation set consisted of 263 molecules for MERS-CoV *M* ^*pro*^ and 297 molecules for SARS-CoV-2 *M* ^*pro*^. Subsequently, we decided to create traditional ML models consisting of RF and XGBoost models in addition to BNN models, with each being a single task regression model for each of the two targets, resulting in six unique models, with the traditional ML models and BNN models being compared upon their predictions for both SARS-CoV-2 *M* ^*pro*^ and MERS-CoV *M* ^*pro*^ respectively.

### Traditional Machine Learning

For the creation of the traditional ML models we utilized the QSPRpred package, which allows the building of Quantitative Structure Property/Activity Relationship (QSPR/QSAR) models.^30^ The training data sets, provided by the organizers of the challenge, containing the pIC_50_ values for the targets MERS-COV *M* ^*pro*^ and SARS-CoV-2 *M* ^*pro*^ were used to build the models.

For each of the targets, a separate single task model was built for the RF^26^ model and XGBoost^25^ model, respectively, resulting in four traditional ML models. For the feature generation, different combinations of Morgan and RDKit Fingerprints^27,29^ were used to obtain the best scoring model (Table S1), which was followed by hyperparameter optimization to obtain the best parameters for each model, resulting in four models, with two models for each of the targets.

The final models were used for the prediction of the validation set. The provided validation sets, consisting of 297 data points for MERS-Cov *M* ^*pro*^ and 263 data points for SARS-CoV-2 *M* ^*pro*^, were used for the predictions. After the challenge concluded, the organizers provided the actual values of the validation set through the Polaris platform, which allowed for an evaluation of the performance of the models (Figure 2). The analysis of the actual pIC_50_ values to the ones predicted by the models shows that during the prediction of the MERS-Cov *M* ^*pro*^ data points the pIC_50_ values were focused in the range of 4–6 for both the XGBoost and RF models, whereas for the SARS-CoV-2 *M* ^*pro*^ prediction the predicted values are distributed more equally between the pIC_50_ ranges of 4–8.

**Figure 2:**
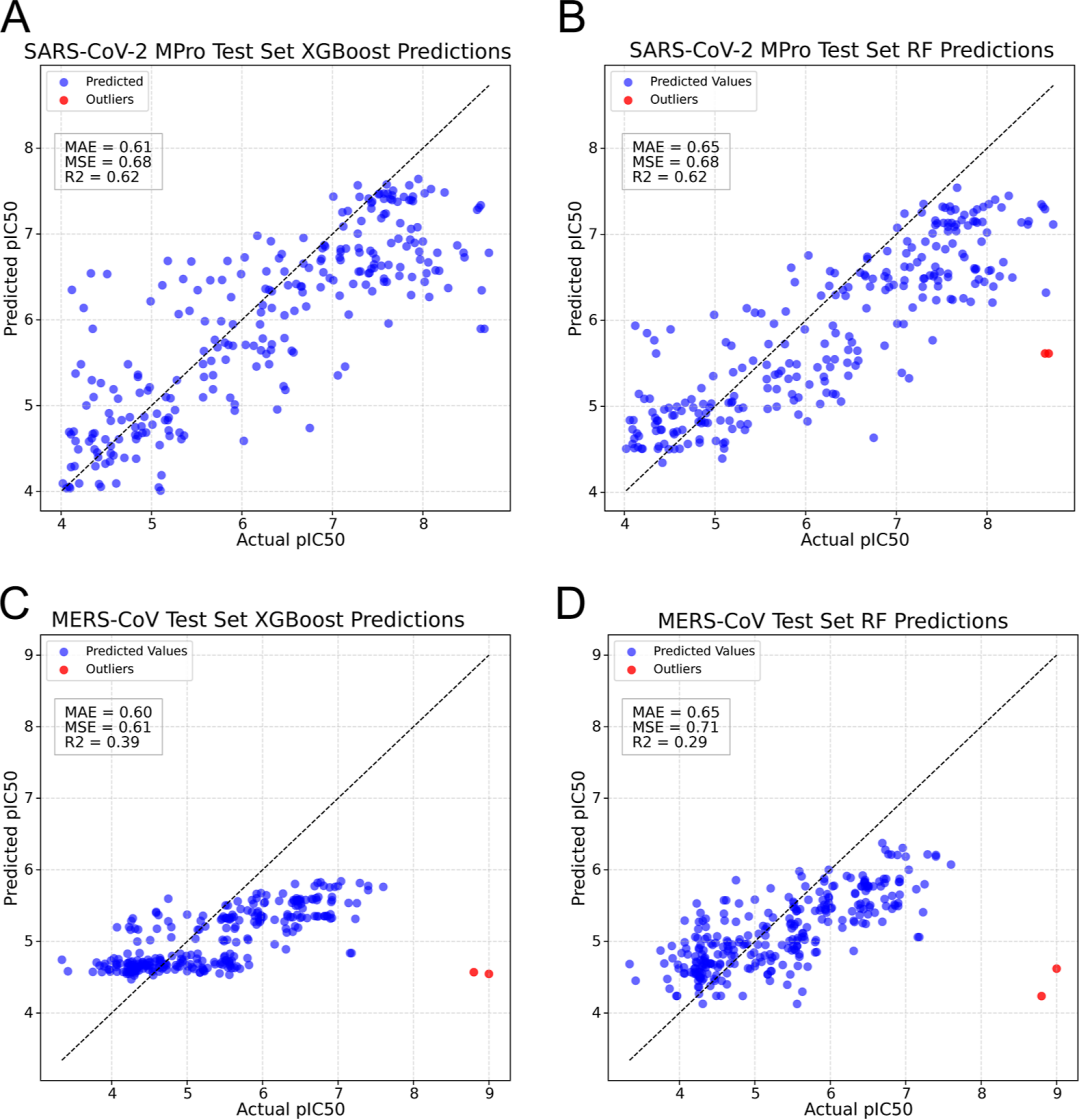
Evaluation of the ML models. (A,B) Actual SARS-CoV-2 *M* ^*pro*^ pIC_50_ values (*x* axis) plotted against predicted pIC_50_ values (*y* axis) of (A) XGBoost and (B) RF models. (C,D) Actual MERS-CoV *M* ^*pro*^ pIC_50_ values (*x* axis) plotted against predicted pIC_50_ values (*y* axis) of (A) XGBoost and (RF) models. Outliers are marked in red.

The results of the predictions of the models were evaluated by using mean average error (MAE), mean squared error (MSE), and *R* ^2^ values. The model performances of the XGBoost and RF models for SARS-CoV-2 *M* ^*pro*^ predictions showed that the XGBoost model performed better than the RF model in absolute error (XGBoost MAE: 0.61, RF MAE: 0.65) but equal in squared error (MSE 0.68) and correlation (*R* ^2^: 0.62). A similar analysis was made for the models that were used for predicting MERS-CoV *M* ^*pro*^. There, the XGBoost model (MAE: 0.60, MSE: 0.61, *R* ^2^: 0.39) outperformed the RF model (MAE: 0.65, MSE: 0.71, *R* ^2^: 0.29) noticeably, showing that for this data set the XGBoost model is more favorable.

### Bayesian Neural Network

The trained BNN models demonstrated differing levels of predictive capability across the two data sets. During inference, we employed 5000 forward passes, allowing us to approximate the posterior predictive distribution and thereby obtain an estimate of both the predictive mean and the associated uncertainty. For the SARS-CoV-2 *M* ^*pro*^ data set, the model achieved an MSE of 0.55, MAE of 0.55, and an *R* ^2^ value of 0.69, indicating reasonably good agreement between predicted and observed values. In contrast, performance on the MERS-CoV *M* ^*pro*^ data set was slightly weaker, with an MSE of 0.58, MAE of 0.56, and an *R* ^2^ of 0.43. It is important to note that due to the random sampling nature of model weights during inference, subsequent evaluation runs of the model may produce slightly different overall outcomes. However, if enough forward passes are conducted, similar values should emerge.

A general pattern we observed during inference was that the mean prediction accuracy improved as the number of forward passes increased. This is consistent with the expected effect of Monte Carlo approximation of the posterior predictive distribution, where a larger sample size of weight realizations yields a more stable and representative estimate of the predictive mean. However, the BNN’s stochastic weight sampling adds randomness to the outputs, so not every observed improvement reflects a shift toward statistical convergence.

Interestingly, while MSE and MAE were similar between the two models, the *R* ^2^ values showed notable differences, indicating that the BNN approach performed better on the SARS-CoV-2 *M* ^*pro*^ data set. Examining the distribution of predictions (Figure 3A,B), the SARS-CoV-2 *M* ^*pro*^ validation set displays a clear trend and a well-spread distribution across predicted activities, whereas the MERS-CoV *M* ^*pro*^ predictions are clustered tightly between 4.5 and 6.

**Figure 3:**
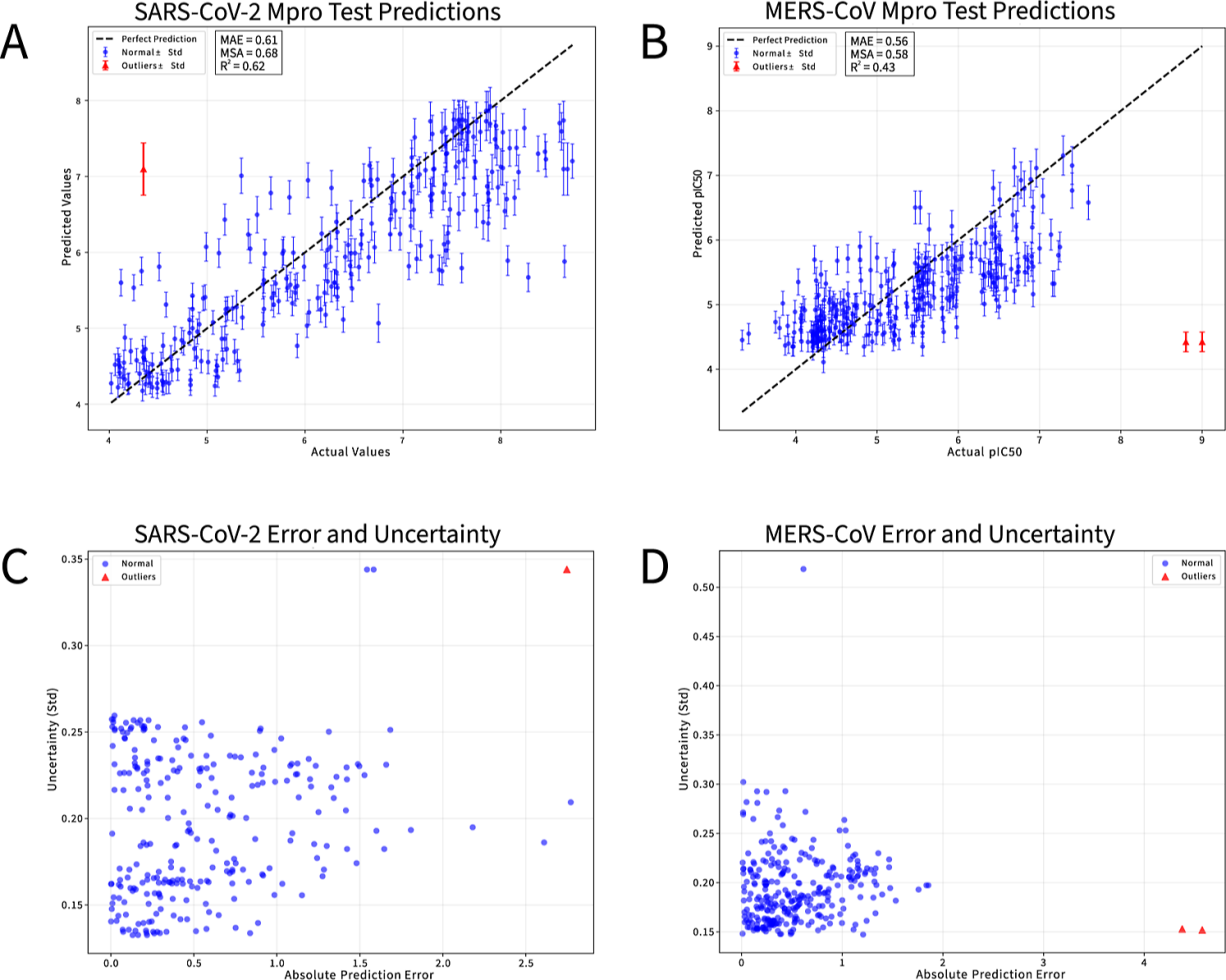
Evaluation results of the BNN. (A,B) Observed pIC_50_ values (*x* axis) plotted against predicted pIC_50_ values (*y* axis) of the validation set from (A) SARS-CoV-2 *M* ^*pro*^ and (B) MERS-CoV *M* ^*pro*^. Outliers are marked in red. (C,D) Absolute prediction error (*x* axis) plotted against the standard deviation (*y* axis) calculated from the 5000 forward passes of each data point of the validation set for (C) SARS-CoV-2 *M* ^*pro*^ and (D) MERS-CoV *M* ^*pro*^. The standard deviation is used as an estimation of the model’s uncertainty of the predicted values.

Furthermore, when comparing prediction errors with the BNNs’ estimated uncertainties (Figure 3C,D), the SARS-CoV-2 *M* ^*pro*^ model successfully flags its main outlier as uncertain, whereas the MERS-CoV *M* ^*pro*^ model predicts its outliers with high confidence despite large errors. Decomposing the BNNs’ total uncertainty into epistemic (model) and aleatoric (data) uncertainties^31^ reveals that both models exhibit substantial total uncertainty, with pIC_50_ variances of 1.02 for MERS and 1.39 for SARS. Notably, the majority of this uncertainty is aleatoric, accounting for 93.1% (pIC_50_ 0.95) in the MERS model and 91.4% (pIC_50_ 1.27) in the SARS model. This suggests that the primary source of uncertainty arises from the data itself rather than the model, which is expected for bioactivity data sets of this type.

### Comparison

When comparing the predictive performance of the traditional machine learning models with the BNN model, several trends are noticeable. These trends can be tied to the underlying data distributions, as well as the different models’ respective architectures. The uncertainty was assessed through nonparametric paired bootstrapping using 1000 resamples.

For the SARS-CoV-2 *M* ^*pro*^ data set, the BNN outperformed both the RF and XGBoost algorithms­(Table 1). The BNN produced more evenly distributed predictions across the observed pIC_50_ range (Figure 3A,B), while the traditional ML models tended to perform worse, especially within higher pIC_50_ ranges (Figure 2A,B). In contrast, the MERS-CoV *M* ^*pro*^ data set presented a greater challenge for all models. Both RF and XGBoost predictions showed clustering in the pIC_50_ range between 4 and 6 (Figure 2C,D), reflecting the models’ tendency to regress toward a mean. While the BNN also exhibited clustering, it predicted a greater number of points outside the dominant range (Figure 3), introducing more variability in its outputs, showing that it had adapted better to the data set.

**Table 1:** Model Performance on SARS-CoV-2 *M* ^*pro*^ and MERS-CoV *M* ^*pro*^

	SARS-CoV-2 *M* ^*pro*^			MERS-CoV *M* ^*pro*^		
model	MAE	MSE	*R* ^2^	MAE	MSE	*R* ^2^
XGBoost	0.61 ± 0.03	0.68 ± 0.07	0.62 ± 0.04	0.60 ± 0.03	0.61 ± 0.10	0.39 ± 0.07
RF	0.65 ± 0.03	0.68 ± 0.07	0.62 ± 0.04	0.65 ± 0.03	0.71 ± 0.10	0.29 ± 0.06
BNN	0.55 ± 0.03	0.55 ± 0.06	0.69 ± 0.04	0.56 ± 0.03	0.58 ± 0.10	0.43 ± 0.07

Across all three models, predictive performance was consistently better for the SARS-CoV-2 *M* ^*pro*^ data set than for MERS-CoV *M* ^*pro*^. In the MERS data set, all models struggled to predict pIC_50_ values further from the mean, even though more data points were available for training. However, when looking at the distribution of the training data set pIC_50_ values, we observe a pronounced imbalance, where most compounds fall within a narrow pIC_50_ range of 4–5 compared to the rather evenly distributed SARS-CoV-2 *M* ^*pro*^ data set (Figure 4). This skew encouraged regression toward the mean, producing the clustering seen in both traditional ML and BNN predictions for MERS-CoV *M* ^*pro*^ data (Figures 3B and 2C,D) and reducing accuracy for less common high- or low-activity cases.

**Figure 4:**
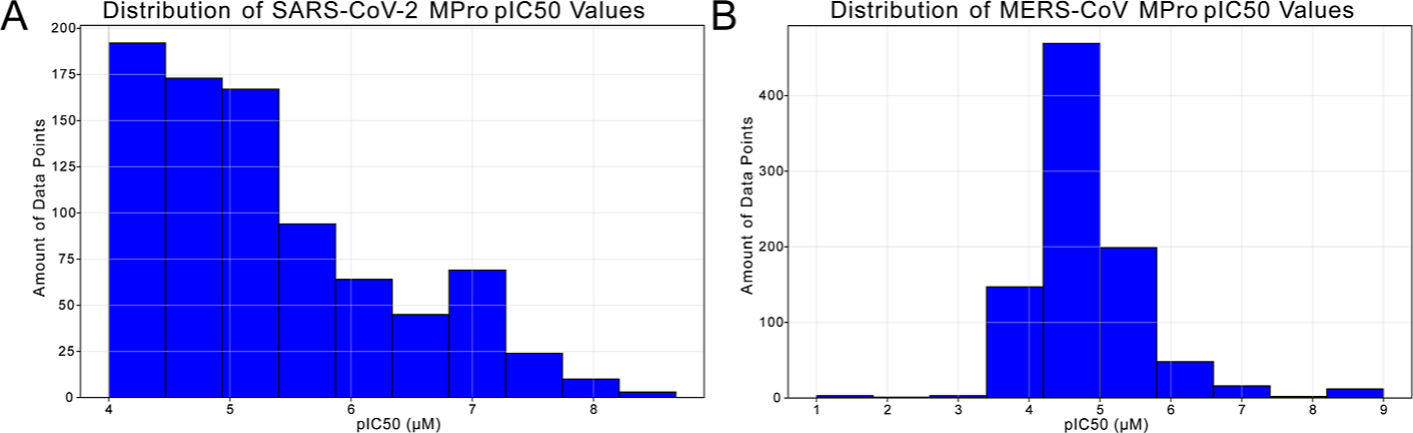
Distribution of pIC_50_ values within the training sets. (A) Shows the distribution of values within the SARS-CoV-2 *M* ^*pro*^ data set, which is relatively balanced. (B) Shows the distribution of values within the MERS-CoV *M* ^*pro*^ data set, where we can see a strong clustering of values between pIC_50_ values of 4 to 5.

Interestingly, all models showed the same two notable outliers within the MERS-CoV data set prediction, whose behavior becomes clearer when examined through principal component analysis (PCA)^32^ of the chemical feature space (Figure 5). The MERS outlier lies in a region of high point density (Figure 5) yet exhibits an unusually high pIC_50_, indicative of an activity cliff. Such minimal structure–activity relationships are notoriously difficult for fingerprint-based models to capture and remain a general challenge in ligand-based machine learning.^33^ Its location within a densely populated region further led the BNN to assign high confidence to an incorrect prediction (Figure 3D). In contrast, the BNNs SARS-CoV-2 data set outlier was mispredicted by the BNN but accompanied by high uncertainty, likely due to the relative sparsity of data in its local chemical space, also seen when comparing the outlier to its nearest neighbors (Figure 5A). These PCA/prediction patterns were consistent across BNN, XGBoost, and RF, with both data sets showing clustering in specific chemical space regions, underscoring how distributional biases in the training data and the choice of molecular descriptor strongly influence predictive performance.

**Figure 5:**
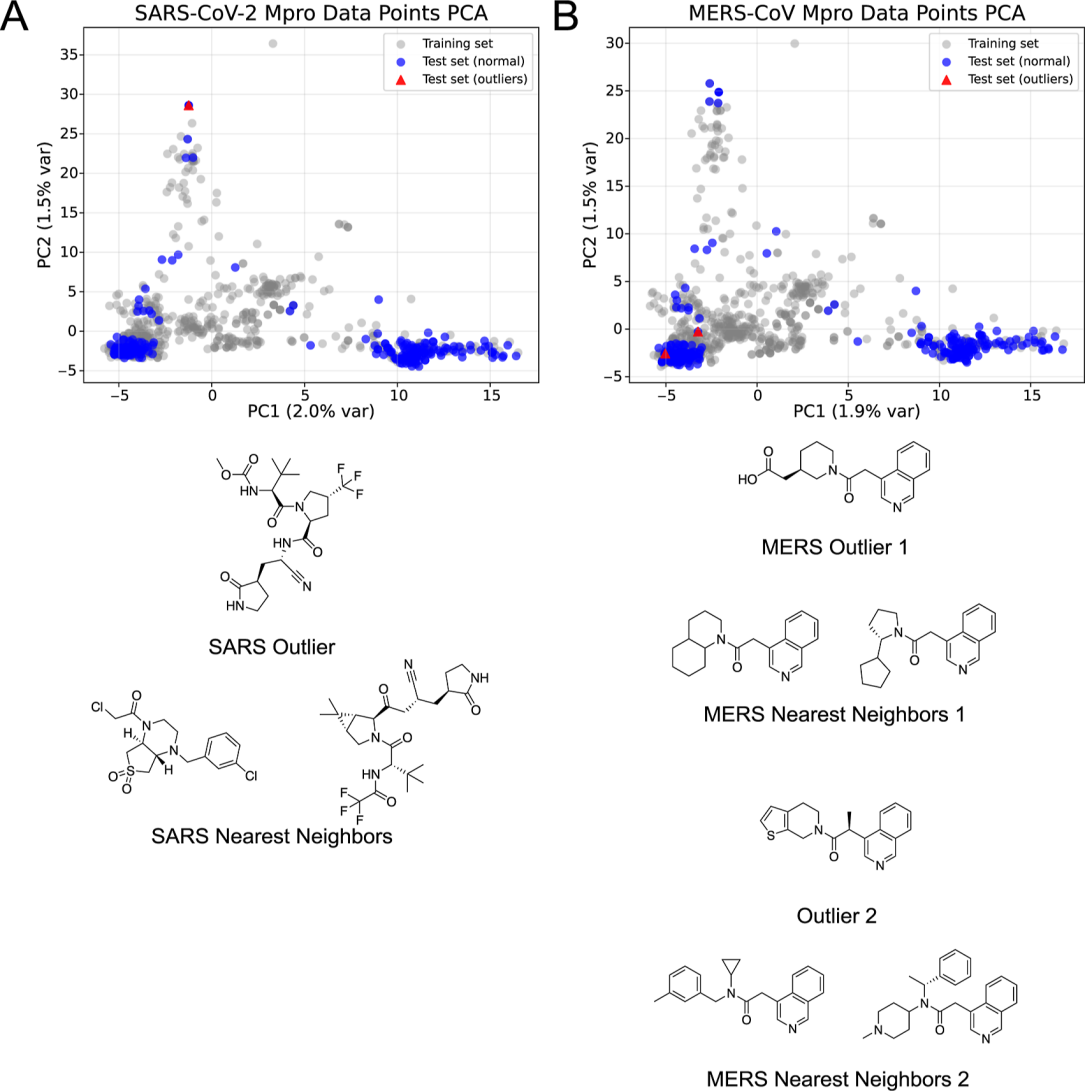
PCA Analysis of SARS-CoV-2 *M* ^*pro*^ MERS-CoV *M* ^*pro*^ with highlighting of the outliers during prediction. (A) PCA analysis of SARS-CoV-2 *M* ^*pro*^ data points, with the one outlier identified in the BNN prediction highlighted. The chemical structure of the outlier, accompanied by its two nearest neighbors, is depicted below. (B) PCA analysis of MERS-CoV *M* ^*pro*^ data points with the two outliers identified in the BNN and ML prediction highlighted. Chemical structures of the outliers, accompanied by their respective two nearest neighbors, are depicted below.

In general, we saw that the predictive models tend to perform best when the underlying activity data is evenly distributed across the target range pIC_50_, as this allows them to learn both high- and low-activity regions without biasing toward the mean. Within our evaluation, the BNN outperformed the traditional RF and XGBoost models in the SARS case, benefiting from its more complex neural network architecture, which may capture nonlinear relationships in the data more effectively. The Bayesian treatment of the network weights further enhances this advantage, as it allows the BNN to adapt to smaller data sets, such as the one obtained during the challenge here, by incorporating uncertainty into the learning process, even though conventional neural networks typically require much larger data sets to generalize well. That said, larger and more balanced data sets would be expected to improve the performance of all models, regardless of architecture. The PCA analyses (Figure 5) reinforce this point by showing that predictive uncertainty (Figure 3) is closely tied to the position of the test compound within the seen chemical feature space, with sparse regions prompting higher uncertainty estimates from the BNN. However, we also observed that denser regions can still lead to overconfident errors as with the MERS data, prompting the question of how to adequately predict activity cliffs.

## Discussion

A problem we noticed during the analysis of the outliers is that the representation of data points fed to the model may have a significant impact on the predictive outcome. The fingerprint-based representations used here, while effective for capturing general structural patterns, may miss subtle features that drive differences in activity, particularly in cases of activity cliffs. Incorporating alternative or complementary descriptor types, such as graph-based embeddings or three-dimensional conformational descriptors, could allow models to capture more nuanced SAR. This could improve both prediction accuracy and uncertainty estimation, especially for challenging data sets such as MERS-CoV *M* ^*pro*^.

While our study primarily explored single-task regression models to predict activity toward the different coronaviruses independently, future work could explore the use of multitask learning approaches. Multitask models have the potential to leverage shared representations across related tasks, thereby improving predictive performance by utilizing a larger pool of available data. This approach could be particularly beneficial when tasks are biologically correlated, as in the case of these two closely related coronavirus main proteases. However, implementing multitask learning introduces additional challenges, particularly in handling incomplete data sets where certain end points are missing for a subset of compounds.

In our models, stereochemical information was not explicitly considered. During data preparation, stereochemical annotations were removed when converting molecules to canonical SMILES, and the subsequent training relied on 2D fingerprints that do not encode chirality. While this strategy simplifies the workflow and allows for consistent baseline comparisons, it also means that stereoisomers, often differing in biological activity, are treated as identical. Consequently, variations in IC_50_ values among compounds sharing the same canonical SMILES may partly reflect unaccounted stereochemical differences. Incorporating chirality-aware descriptors in future work could improve predictive accuracy, although for the relatively small data sets examined here, such detailed representations might also increase the risk of model overfitting.

It should be acknowledged that, given the limited data sets, these findings cannot be readily generalized across all targets. Nevertheless, BNNs are inherently well-suited for such settings, as they can learn hierarchical features directly from the data while providing better-calibrated predictive distributions. Notably, a substantial portion of the BNN’s uncertainty is aleatoric, reflecting the noise in the experimental data. This highlights the network’s ability to recognize and accommodate noisy inputs, rather than overfitting to them. Such probabilistic awareness not only contributes to the observed predictive performance but also supports more informed decision-making under resource constraints. While BNNs generally require longer training times compared to conventional models, this is less of a concern when dealing with relatively small data sets, as in the present study. In medicinal chemistry studies, where experimental testing is costly, this uncertainty enables prioritization of compounds with novel scaffolds that were not covered by the training set, while still leveraging knowledge from available data, effectively balancing exploration of new chemical space with exploitation of known patterns.

In our case, the BNNs outperformed the tree-based approaches likely because the training data comprised only a few hundred compounds with considerable noise in both structural diversity and activity measurements. Under these conditions, the BNN’s capacity to represent parameter uncertainty may have helped prevent overfitting to sparse or noisy regions of chemical space. Moreover, the intrinsic ability of BNNs to quantify predictive uncertainty is particularly valuable in drug discovery campaigns, as it enables more informed compound prioritization by highlighting predictions made with higher confidence.

During the challenge, intermediate models were submitted on the Polaris platform.^9^ During the first intermediate submission, a preliminary XGBoost was used for the prediction of MERS-CoV *M* ^*pro*^ potency, while BNN was used for the SARS-CoV-2 *M* ^*pro*^ potency prediction. During the second intermediate submission, the prediction for SARS-CoV-2 *M* ^*pro*^ was exchanged for a XGBoost model prediction to obtain results from the organizers, allowing us to verify our assumptions that the BNN model may perform better. The results showed that the preliminary BNN model performed better for SARS-CoV-2 *M* ^*pro*^ than the preliminary XGBoost model. The approach following the second intermediate results was to improve the BNN models and apply BNN models for both the prediction of SARS-CoV-2 *M* ^*pro*^ and MERS-CoV *M* ^*pro*^ for the final submission. During the timeline between the first intermediate submission and the final submission, we encountered technical issues, which limited our capacities for model generation, thus allowing only for the generation of preliminary ML models, which resulted in our inability to pursue the improvement of the models during the timeline of the challenge. The predictions submitted during the challenge placed us on 16th place. This is a satisfying result considering the technical hindrance encountered during the challenge. Nonetheless, it would have been great to have been able to improve and get the final BNN model used for evaluation in this work during the challenge.

The nature of the challenge allowed us to test out the models, which was possible due to the efforts of the organizers of the challenge, who were in contact with the participants during the challenge with the Polaris platform being used for the submission of the predictions. The intermediate results, provided by the organizers of the challenge, were a convenient way to evaluate the predictions and thus allowed for an adjustment and improvement of the models.

## Conclusion

In this study, we applied BNN, RF, and gradient boosting (XGBoost) to the SARS-CoV-2 and MERS-CoV *M* ^*pro*^ data sets provided by the Polaris ASAP Discovery potency prediction challenge 2025. Overall, the BNN consistently outperformed the traditional machine learning models, even in sparse or unevenly distributed data regimes, highlighting its ability to capture complex, nonlinear relationships while allowing uncertainty estimation. Looking forward, BNNs could become particularly powerful when combined with more modern or complementary chemical descriptors, potentially enhancing both predictive accuracy and reliability. That said, traditional ML approaches such as RF and gradient boosting remain valuable as robust baseline methods and should not be overlooked in early stage ligand-based modeling.

## Methods

### Data Preparation

The data set was originally retrieved from the Polaris Asap-Discovery antiviral potency 2025 challenge.^9^ The initial training data set used to predict coronavirus protease inhibition potency comprised 1031 molecules with reported pIC_50_ values for SARS-CoV-2 M^pro^ and MERS-CoV M^pro^. Some molecules contained data for both targets, while others were associated with only one. In total, the data set included 901 data points for SARS-CoV-2 M^pro^ and 842 for MERS-CoV M^pro^. The data sets for training were prepared by extracting CXSMILES and pIC_50_ values. The CXSMILES were then converted to canonical SMILES and salts were washed out using the RDKit SaltRemover.^27^ This conversion removed information on stereoisomerism, resulting in duplicate molecules. To retain information about potential IC_50_ differences resulting from stereoisomers, the duplicates were kept. The SMILES representations were converted into molecular fingerprints using RDKit^27^ to enable effective model training. The 2D fingerprints used did not encode stereochemical information. During hyperparameter optimization of the models, we employed several types of fingerprints: Morgan fingerprints with a radius of either 2 or 3 and bit lengths of 1024 or 2048, as well as RDKit fingerprints using maximum path lengths of 5, 6, 7, or 8 and bit lengths of 256, 512, or 1024.^29^ We also tested a combination of both fingerprint types, concatenating Morgan fingerprints (radius 2 or 3, 512, or 1024 bits) with RDKit fingerprints (max path lengths of 5–8 and 256, 512, or 1024 bits) to capture complementary features.

### Model Training

#### Traditional Machine Learning Models

The traditional machine learning methods, consisting of the gradient boosting model and the random forest model, were implemented using the *QSPRpred* package.^30^ For the prediction of the SARS-CoV-2 *M* ^*pro*^ and MERS-COV *M* ^*pro*^ potency, two separate single task regression models were created for both the gradient boosting model and the random forest model respectively. For each of the models, a cluster split^34^ functionality that is present in *QSPRpred* package^30^ and utilizes tanimoto similarity of fingerprints and min–max seeding was applied to split the data set into a training set consisting of 80% of the data set and a test set consisting of the remaining 20% of the data set. For the traditional machine learning models, during the feature generation, low variance features (variance <0.001) were removed and the retained features were standardized using the StandardScaler function present in *sklearn*.^34^ The final models were trained using the full data set.

The hyperparameter optimization with a selection of parameters (Tables S2 and S3) consisted of two steps. The first step was focusing on identifying the best complementary features as previously described. For this the *Optuna* ^35^ framework with 300 trials was used for all of the models. Both the SARS-CoV-2 *M* ^*pro*^ and MERS-CoV *M* ^*pro*^ potency prediction models showed the best results with the combination of the Morgan fingerprints and RDKit fingerprints (Table S1).^27,29^ The second step was focused on the hyperparameter optimization with the selected best performing feature combination. For this step, the *Optuna* ^35^ framework with 500 trials for the random forest models and 2500 trials for the XGBoost models was used, resulting in the identification of the optimal parameters for the models (Tables S4 and S5). The negative mean average error was used as a scoring function in both steps of the hyperparameter optimization.

During the hyperparameter optimization of the final models, XGBoost models achieved MAE values of 0.52895091 and 0.4315248 for the predictions for SARS-CoV-2 *M* ^*pro*^ and MERS-CoV *M* ^*pro*^, while the RF models achieved values of 0.61800179 and 0.48992966 respectively. The complete process of the hyperparameter optimization and final model training consisted of 4.1 h for MERS-CoV *M* ^*pro*^ and 2.0 h for SARS-CoV-2 *M* ^*pro*^ for the RF models and 59.7 h for MERS-CoV *M* ^*pro*^ and 44.8 h for SARS-CoV-2 *M* ^*pro*^ for the XG models. 32 CPU cores were used in parallel during the optimization and training. Final inference on the validation set, required approximately 0.007 s per molecule.

#### Bayesian Neural Network

Bayesian neural network models were implemented using *PyTorch 2.4.1* ^36^ in combination with *torchbnn 1.2*.^24^ Separate models were trained to predict the biological activity of compounds against SARS-CoV-2 Mpr and MERS-CoV *M* ^*pro*^ coronaviruses. The input layer size matched the length of the molecular fingerprint used, and was followed by zero to three hidden layers, with layer sizes chosen from 64, 128, 256, 512, and 1024. The output layer consisted of a single neuron representing the predicted pIC_50_ value. Leaky ReLU activation functions with a negative slope of 0.01 were applied between layers to address the “dying ReLU” problem.^37^ Models were also tested both with and without normalization layers.

Hyperparameter optimization was performed using the *Optuna* ^35^ framework with 10,000 trials being run. The following ranges were explored during optimization: the weighting of the Kullback–Leibler (KL)^38^ divergence and learning rate were sampled from log-uniform distributions spanning [10^–4^, 10^–1^] and [10^–4^, 10^–2^], respectively; the batch size was selected from {32, 64, 128}; dropout rates were drawn from a uniform distribution between 0.0 and 0.5; and the parameters of the prior distribution, namely the prior μ (mean of the prior Gaussian) and prior σ (standard deviation of the prior), were sampled from uniform distributions over [−0.2, 0.2] and [0.01, 0.2], respectively. These hyperparameters were jointly optimized along with different fingerprint inputs, varying hidden layers, and normalization of layers to balance predictive accuracy with Bayesian regularization. The loss function for optimization was defined as a weighted combination of mean squared error (MSE) and mean absolute error (MAE), specifically 0.3 × MSE + 0.7 × MAE, plus a Bayesian regularization term that represents the average Kullback–Leibler divergence between the approximate posterior and prior distributions over the network weights, scaled by a KL weight and the goal was to minimize the loss.

6 $$L=0.3\cdot MSE\left(y,ŷ\right)+0.7\cdot MAE\left(y,ŷ\right)+\lambda_{KL}\cdot \frac{1}{N_{KL}}\sum_{i=1}^{N_{KL}}KL\left(q\left(w_{i}\right)∥p\left(w_{i}\right)\right)$$

where

7 $$MSE\left(y,ŷ\right)=\frac{1}{N}\sum_{i=1}^{N}{\left(y_{i}-{ŷ}_{i}\right)}^{2}$$

8 $$MAE\left(y,ŷ\right)=\frac{1}{N}\sum_{i=1}^{N}\left|y_{i}-{ŷ}_{i}\right|$$

and

9 y=truetargetvalues

10 ŷ=predictedtargetvalues

11 $$\lambda_{KL}=KL\;weight$$

12 $$N_{KL}=number\;of\;KL\;divergence\;terms\;\left(e.g.,number\;of\;weight\;parameters\right)$$

13 $$KL\left(q\left(w_{i}\right)∥p\left(w_{i}\right)\right)=KL\;divergence\;between\;the\;approximate\;prior\;\text{and}\;posterior\;of\;the\;weight\;w_{i}$$

During hyperparameter optimization, each trial was evaluated using 5-fold cross-validation with an 80/20 random train-test splits. The average loss across all folds is used to assess the performance of the hyperparameter combination and select the optimal hyperparameters. The final model was then trained on the entire data set using the best identified combination of hyperparameters, allowing the model to leverage all available data points, given the relative sparsity of data. The final hyperparameters for training were as follows: for the SARS model, fingerprint type was set to a combination of morgan and RDKit fingerprints^27,29^ with a radius of 3 and 512 bits for the morgan fingerprint, while the RDKit fingerprint parameters were a maximum path of 7 and 1024 bits; the network comprised three hidden layers with dimensions 256, 1024, and 64, the weighting of the KL divergence set to 1.2739 × 10^–4^, a learning rate of 6.7605 × 10^–3^, a batch size of 128, a dropout rate of 1.1159 × 10^–1^, a prior μ of 1.6414 × 10^–2^, a prior σ of 1.0082 × 10^–1^, and layer normalization. For the MERS model, the fingerprint was soley a Morgan fingerprint with a radius of 2 and 2048 bits; the architecture had three hidden layers, with dimensions 512, 128, and 512, a KL weight of 9.9644 × 10^–4^, a learning rate of 1.9114 × 10^–3^, a batch size of 32, a dropout rate of 4.6384 × 10^–1^, a prior μ of 4.5296 × 10^–2^, a prior σ of 6.9908 × 10^–2^, and no normalization. A single model generation for the BNN took ∼15 s making the total hyperparameter optimization and final model training take ∼46.6 h computing on NVIDIA RTX3090 GPUs. Final inference on the validation set, using 5000 forward passes, required approximately 1 s per molecule.

#### Equation

For the evaluation of the individual models, multiple metrics were used, including MSE, MAE and *R* ^2^, calculated as follows

14 $$MSE\left(y,ŷ\right)=\frac{1}{N}\sum_{i=1}^{N}{\left(y_{i}-{ŷ}_{i}\right)}^{2}$$

15 $$MAE\left(y,ŷ\right)=\frac{1}{N}\sum_{i=1}^{N}\left|y_{i}-{ŷ}_{i}\right|$$

16 $$R^{2}=1-\frac{\sum_{i=1}^{n}{\left(y_{i}-{ŷ}_{i}\right)}^{2}}{\sum_{i=1}^{n}{\left(y_{i}-\mathrm{y̅}\right)}^{2}}$$

where

17 y=truetargetvalues

18 ŷ=predictedtargetvalues

Outliers were in the prediction plots were identified using the zscore within SciPy.^39^ Every data point with a zscore higher than 4 was flagged as an outlier. The robustness of the model metrics for the validation set (MAE, MSE and *R* ^2^) was assessed via nonparametric paired bootstrap resampling with 1000 iterations. The test set was treated as n paired observations. We sampled indices with replacement per iteration, recomputed the metrics, and summarized the resulting distributions as mean ± standard deviation.

The principal component analysis of the data sets was done based on Morgan fingerprints with radius 3 and 2048 bits generated using RDKit.^27^ Two principal components were then calculated using scikit-learn^34^ and based on the combined training and validation sets, with the plots being generated using matplotlib.^40^ Nearest neighbors were calculated based on the Euclidean distance between fingerprints.

The total predictive uncertainty in Bayesian Neural Networks (BNNs) was first estimated from the standard deviation of predictions obtained across multiple Monte Carlo forward passes. This uncertainty was then decomposed into *epistemic* and *aleatoric* components.^31^

The epistemic uncertainty captures uncertainty in the model parameters and is estimated via Monte Carlo sampling from the posterior distribution. Specifically, for *T* stochastic forward passes using Monte Carlo dropout, we obtain predictions ${\left\{{ŷ}_{t}\right\}}_{t=1}^{T}$ , and the epistemic uncertainty is quantified as the variance of the mean predictions

19 $$\sigma_{epistemic}^{2}=Var\left(E\left[{ŷ}_{t}|x,w_{t}\right]\right)$$

20 $$where\;w_{t}\;denotes\;the\;sampled\;network\;weights.$$

The aleatoric uncertainty, by contrast, reflects the inherent noise in the data and is modeled explicitly in heteroscedastic networks through a separate variance head that predicts a data-dependent variance *σ*_data_^2^(**x**) for each input **x**. The aleatoric component is then computed as the expected value of these predicted variances across Monte Carlo samples

21 $$\sigma_{aleatoric}^{2}=E\left[\sigma_{data}^{2}\left(x\right)\right]$$

Finally, the total predictive uncertainty combines both contributions

22 $$\sigma_{total}^{2}=\sigma_{epistemic}^{2}+\sigma_{aleatoric}^{2}$$

In our implementation, *T* = 1000 Monte Carlo samples were used to approximate these expectations, with dropout serving as the stochastic mechanism for parameter sampling from the approximate posterior.

## Supplementary Material



## Data Availability

The full data set is available through the Polaris ASAP Discovery antiviral potency prediction challenge (https://polarishub.io/datasets/asap-discovery/antiviral-potency-2025-unblinded). The scripts used for training, evaluation, and prediction and final models are available through the wolberlab GitHub (https://github.com/wolberlab/polaris_antiviral_challenge). Coding assistance was provided by *ChatGPT* (OpenAI, L.L.C., San Francisco, CA), *Claude* (Anthropic PBC, San Francisco, CA), and *GitHub Copilot* (GitHub Inc., San Francisco, CA). Grammar, spelling, punctuation, and tone were edited with *ChatGPT*, *Claude*, and *DeepL* (DeepL SE, Cologne, Germany).

## Notes

S1–S5: ML model hyperparameters, S6: distribution of chiral molecules within the training sets, S7: UMAP representation of the chemical space for the MERS and SARS models (PDF)
