# Supplementary material for: Fingerprint-Based Machine Learning for SARS-CoV‑2 and MERS-CoV M pro Inhibition: Highlighting the Potential of Bayesian Neural Networks
Source: J Chem Inf Model. 2025 Dec 10;65(24):13255–65. doi: 10.1021/acs.jcim.5c02014 (PMC12728926; doi:10.1021/acs.jcim.5c02014)
Supplement: Supplementary file 1 [file ci5c02014_si_001.pdf]

Supporting Information for Fingerprint-Based Machine Learning for SARS-CoV-2 and MERS-CoV M_{pro} Inhibition: Highlighting the Potential of Bayesian Neural Networks

Niklas Piet Doering,[†,‡] Valerij Talagayev,[‡,†] Sijie Liu,[‡] and Gerhard Wolber[*,‡]

†*equally contributed to this work*

‡*Freie Universität Berlin, Department of Biology, Chemistry and Pharmacy, Institute of Pharmacy, Molecular Design Group, Königin-Luisestr. 2+4, 14195 Berlin, Germany*

E-mail: gerhard.wolber@fu-berlin.de

Table S1: Selected fingerprints and models for SARS-CoV-2 Mpro and MERS-CoV Mpro potency predictions.

**Virus**	**Model**	**FP Type**	**Radius / MaxPath**	**nBits**
SARS-CoV-2 Mpro	XGBoost	MorganFP	3	1024
		RDKitFP	5	256
	Random Forest	MorganFP	3	1024
		RDKitFP	4	256
MERS-CoV Mpro	XGBoost	MorganFP	3	1024
		RDKitFP	4	256
	Random Forest	MorganFP	3	1024
		RDKitFP	4	256

Table S2: XGBoost parameters optimized during hyperparameter tuning with their respective value ranges.

**Parameter**	**Min**	**Max**
max_depth	1	20
gamma	0	20
max_delta_step	0	20
min_child_weight	1	20
learning_rate	0.001	1
subsample	0.001	1
n_estimators	10	250

Table S3: RF parameters optimized during hyperparameter tuning with their respective value ranges.

Parameter	Min	Max
n_estimators	1	20
max_depth	0	20
min_sample_split	0	20
min_samples_leaf	1	20
**Parameter**	**Values**	
max_features	sqrt, log2, None	

Table S4: XGBoost final model hyperparameters for SARS-CoV-2 Mpro and MERS-CoV Mpro potency prediction.

**Hyperparameter**	**SARS-CoV-2 Mpro**	**MERS-CoV Mpro**
max_depth	11	14
gamma	0	0
max_delta_step	14	8
min_child_weight	14	18
learning_rate	0.1043	0.1830
subsample	0.3883	0.3060
n_estimators	101	18

Table S5: RF final model hyperparameters for SARS-CoV-2 Mpro and MERS-CoV Mpro potency prediction.

**Hyperparameter**	**SARS-CoV-2 Mpro**	**MERS-CoV Mpro**
n_estimators	18	93
max_depth	31	14
min_sample_split	14	20
min_samples_leaf	1	12
max_features	sqrt	sqrt

Table S6: Distribution of chiral molecules within the training sets

	**Molecules**	**Chiral Molecules**	**Enantiomers**	**Stereoisomers**
**SARS**	901	623	58	149
**MERS**	842	648	71	173

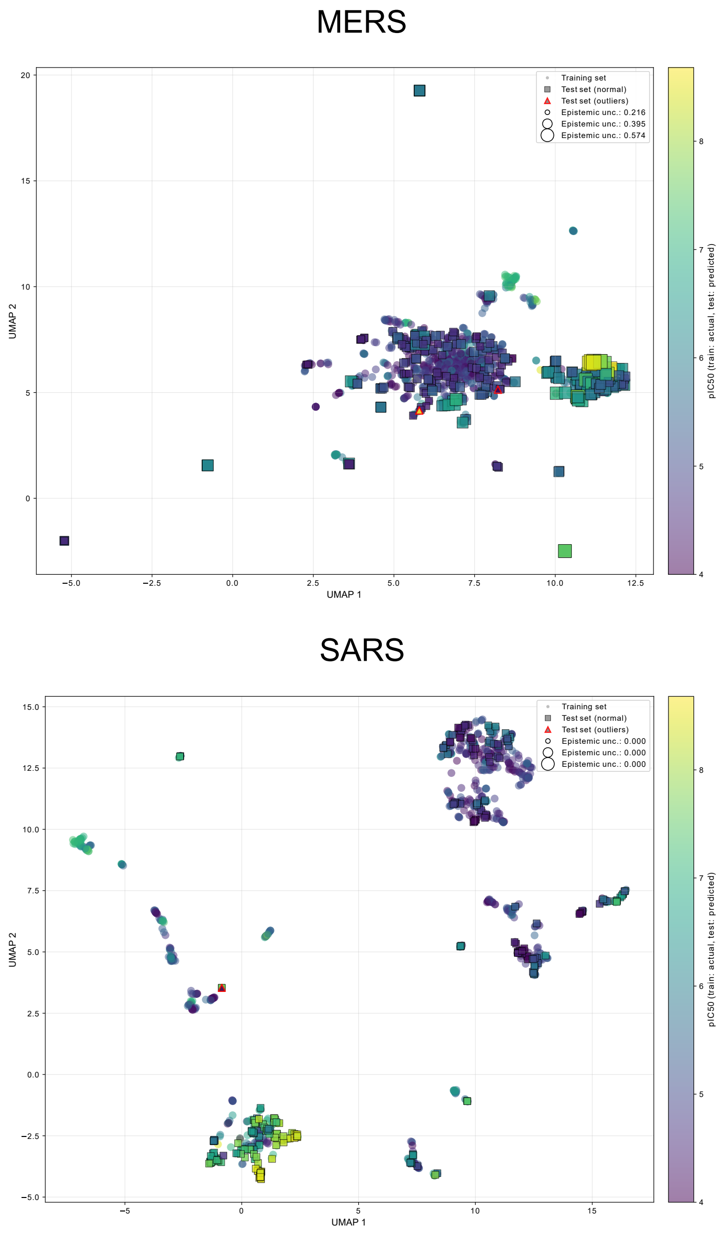

Figure S7: **UMAP representation of the chemical space for the MERS and SARS models.** The training set is shown as small background dots, colored according to experimental IC_{50} values. Test/validation compounds are represented as squares, where color indicates the predicted IC_{50} and size reflects the epistemic uncertainty. Outliers in prediction are highlighted as red triangles. For the MERS model, the outliers lie at the edge of a high-density region, consistent with the PCA projection and explaining why these compounds were confidently mispredicted. Interestingly, the cluster on the right exhibits high epistemic uncertainty, likely because the model is less confident when extrapolating beyond the main data distribution. In the SARS model, by contrast, the outlier appears in a low-density region, which plausibly accounts for its higher predictive uncertainty.
